# Fc receptor-like 5 (FCRL5)-directed CAR-T cells exhibit antitumor activity against multiple myeloma

**DOI:** 10.1038/s41392-023-01702-2

**Published:** 2024-01-12

**Authors:** Zhengyu Yu, Hexian Li, Qizhong Lu, Zongliang Zhang, Aiping Tong, Ting Niu

**Affiliations:** Department of Hematology, State Key Laboratory of Biotherapy and Cancer Center, West China Hospital, Sichuan University, Chengdu, 610041 China

**Keywords:** Haematological cancer, Haematological cancer

## Abstract

Multiple myeloma (MM) remains a challenging hematologic malignancy despite advancements in chimeric antigen receptor T-cell (CAR-T) therapy. Current targets of CAR-T cells used in MM immunotherapy have limitations, with a subset of patients experiencing antigen loss resulting in relapse. Therefore, novel targets for enhancing CAR-T cell therapy in MM remain needed. Fc receptor-like 5 (FCRL5) is a protein marker with considerably upregulated expression in MM and has emerged as a promising target for CAR-T cell therapeutic interventions, offering an alternative treatment for MM. To further explore this option, we designed FCRL5-directed CAR-T cells and assessed their cytotoxicity in vitro using a co-culture system and in vivo using MM cell-derived xenograft models, specifically focusing on MM with gain of chromosome 1q21. Given the challenges in CAR-T therapies arising from limited T cell persistence, our approach incorporates interleukin-15 (IL-15), which enhances the functionality of central memory T (TCM) cells, into the design of FCRL5-directed CAR-T cells, to improve cytotoxicity and reduce T-cell dysfunction, thereby promoting greater CAR-T cell survival and efficacy. Both in vitro and xenograft models displayed that FCRL5 CAR-T cells incorporating IL-15 exhibited potent antitumor efficacy, effectively inhibiting the proliferation of MM cells and leading to remarkable tumor suppression. Our results highlight the capacity of FCRL5-specific CAR-T cells with the integration of IL-15 to improve the therapeutic potency, suggesting a potential novel immunotherapeutic strategy for MM treatment.

## Introduction

Multiple myeloma (MM) is a hematological malignancy characterized by aberrant plasma cell proliferation within the bone marrow and overproduction of monoclonal immunoglobulins.^1^ With a steady increase in incidence, MM has emerged as a considerable health challenge in recent years, accounting for 1% of all cancers and 10% of all hematologic malignancies globally.^2^ Over the past two decades, the overall response rate and overall survival of MM patients have been greatly enhanced by the advent of new medications,^3^ including immunomodulatory drugs (IMiDs) like thalidomide, lenalidomide, and pomalidomide; proteasome inhibitors (PIs) like bortezomib, carfilzomib, and ixazomib, as well as histone deacetylase inhibitors. Monoclonal antibodies have ushered in a new era of MM immunotherapy, with the most successful being Daratumumab and Isatuximab, which target CD38,^4,5^ and Elotuzumab, which targets SLAMF7 located on myeloma cells and natural killer cells.^6^ The utilization of these medications has revolutionized the MM treatment paradigm from conventional chemotherapy to a more personalized, precision medicine. However, resistance and toxic effects (cardiac, renal, and pulmonary toxicities, peripheral neuropathy) have become obstacles.^7^ Combination therapy may hold the key to overcoming resistance and improving long-term treatment results. The combination of IMiDs, PIs, and dexamethasone is the standard protocol for patients with relapsed and refractory multiple myeloma (RRMM),^8^ but it comes with an increased risk of toxic effects for multiple drug combinations.^9^ In fact, the majority of patients with MM are still incurable, and almost all of them will eventually relapse. Therefore, when devising the treatment protocol for each relapse, multiple factors need to be taken into account, and a triple therapy incorporating at least two novel drugs should be considered for non-refractory patients.^10^ It can provide more possibility of remission for patients.

Chimeric antigen receptor T (CAR-T) cell therapy, which targets myeloma via a mechanism distinct from previous anti-myeloma agents, has demonstrated extraordinary efficacy in the treatment of RRMM.^11^ The antigen targeted by the CAR can vary depending on the specific CAR-T cell therapy used, thus offering superior efficacy and safety profiles over traditional therapies. Among them, B cell maturation antigen (BCMA)-targeting CAR-T cells (e.g., JNJ-4528 and LCAR-B38M) is the most successful in improving the outcomes of patients.^12^ Based on the promising results observed in the Phase II clinical trial, The United States Food and Drug Administration (FDA) has granted approval for the use of Idecabtagene Vicleucel (ide-cel, bb2121) in 2021, and Ciltacabtagene Autoleucel (cilta-cel) in 2022, two drugs targeting BCMA for the treatment of RRMM. Despite the BCMA-targeted CAR-T cell therapy has achieved unprecedented efficacy in the treatment of RRMM, numerous patients with MM still experience relapse after treatment owing to factors like antigen escape or downregulation, BCMA shedding, CAR-T cell exhaustion, and the intricate tumor microenvironment.^13^ The same problem of targeting antigens with CAR-T therapy is seen in the treatment of other diseases, 30–70% of refractory B-cell lymphoma patients experiencing disease recurrence following CD19-targeted CAR-T cell therapy exhibit downregulation or loss of CD19 antigen expression.^14^ Similarly, a decline in IL13Ra2 expression has been noted in glioblastoma cases post-CAR-T therapy targeting IL13Ra2, indicating the resurgence of the tumor.^15^ Therefore, a successful strategy is to identify different potential therapeutic targets. At present, GPRC5D--targeted CAR-T cell therapy obtained good results. One study of the GPRC5D-targeted CAR-T therapy observed responses at 25 ×10^6^ to 450 ×10^6^ cells dose levels, with 71% of the patients, including those who had relapsed after BCMA-targeted CAR-T cell therapy, showing a response.^16^ All patients in another GPRC5D-targeted CAR-T cell therapy study demonstrated an overall response, with 60% exhibiting a stringent complete response and 40% exhibiting a very good partial response.^17^ However, a cure for MM is still a long way off, as a significant proportion of patients end up with disease recurrence or progression after receiving targeted CAR T cell therapy, despite initially achieving minimal residual disease negative status.^10^ Despite encouraging preliminary results in MM CAR-T clinical trials, some side effects are still unavoidable, including hematological toxic effects such as neutropenia, leukopenia and thrombocytopenia, and cytokine release syndrome, as well as neurological toxic effects.^16–18^ Considering the limitations of current CAR-T targets for MM treatment, it is essential to explore more dependable and secure alternatives.

Fc receptor-like 5 (FCRL5), a surface marker found specifically on plasma cells in myeloma,^19^ has attracted attention as a possible therapeutic approach. FCRL5 is not only continuously expressed in malignant plasma cells (PCs) of patients,^20^ but also promotes B-cell proliferation and isotype expression after exposure to the antigen.^21–23^ Moreover, FCRL5-targeted antibody-drug conjugates are proven to be effective in treating MM.^19,20^ Therefore, FCRL5 is a potential target for CAR-T MM therapy. The efficacy of CAR-T therapies is often constrained by limited T cell persistence, increased apoptotic susceptibility, and suboptimal proliferative capacity, culminating in disease relapse. Therefore, enhancing T-cell persistence via genetic modification offers a prospective solution. For instance, interleukin-15 (IL-15) has shown promise for augmenting the functionality of central memory T (TCM) cells,^24^ and its inclusion in CAR constructs has improved CAR-T cell survival and activity.^25,26^

To explore this possibility, in this study, we observed consistent FCRL5 expression, even in situations where BCMA expression diminished, following the application of BCMA-targeted CAR-T cells. In addition, CAR-T cells specifically targeting FCRL5, along with a variant of CAR-T cells that secretes IL-15 were engineered. Further, their therapeutic potential against MM was evaluated in vitro under co-culture with various MM cell lines and in vivo using cell-derived xenograft mouse models. Moreover, we intend to use more reliable PDX models for validation and demonstrate the therapeutic safety of FCRL5 CAR-T cells. Our study supports a new approach for MM treatment.

## Results

### Evaluation of FCRL5 expression in MM

**Fig. 1:**
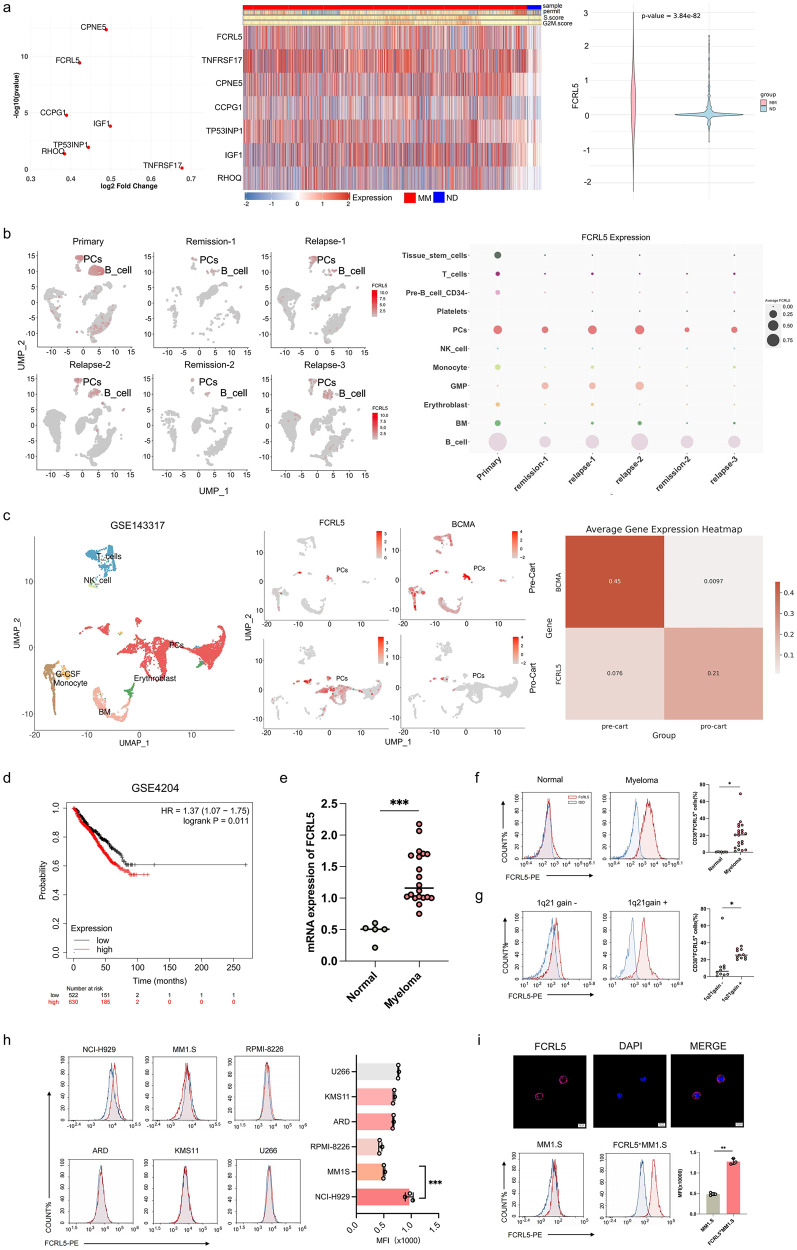
Expression of FCRL5 in multiple myeloma (MM). **a** Volcano and heatmap plots displaying differential gene expression profiles across plasma clusters in six healthy donors and six patients with MM. Log2(fold change) > 1 and *P*adj < 0.05 are considered significant differentially expressed genes. Violin plots of FCRL5 levels from the GSE223060 dataset were generated using two-sided unpaired Wilcoxon signed-rank tests. **b** UMAP of the plasma cells from Patient 27522 at six stages.The UMAP plot illustrates the FCRL5 expression patterns, with color intensity indicating expression levels. The scatter plot illustrates dynamic FCRL5 expression alterations across disease stages, with node size indicating the cluster magnitude. **c** UMAP of bone marrow in patients with MM treated with BCMA CAR-T-cells at pre- and post-CAR-T cell stages, colored by cell type, showing FCRL5 and BCMA expression. **d** Kaplan‒Meier analysis of the GSE4204 dataset; subjects are classified by median module scores, with *P*-values calculated via the log-rank test. **e** Relative mRNA expression of *FCRL5* relative to that of *GAPDH* in normal (*n* = 5) and MM (*n* = 20) samples. **f**, **g** Flow cytometry of FCRL5 expression using an FCRL5-specific monoclonal antibody with gating for FSc/SSc, CD38, and CD45 markers. The results from a representative MM patient and healthy controls are shown (*P < 0.05 vs. normal or 1q21gain- samples). **h** FCRL5 expression in myeloma cell lines assessed by flow cytometry. **i** Immunofluorescence and flow cytometry indicating FCRL5 overexpression in myeloma cells; scale bars = 10 μm (** *P* < 0.01 vs. MM1.S)

As high FCRL5 expression is maintained in PCs from patients,^27^ we first used the Genotype-Tissue Expression (GTEx) datasets and public Human Protein Atlas (HPA)^28^ to evaluate FCRL5 expression in various tissues of individuals and identified FCRL5 expression pattern in tonsils, lymph nodes, spleen, and appendix of lymphoid tissues (Supplementary Fig. S1a). Analysis of the Gene Expression Omnibus (GEO) GSE223060 dataset showed elevated FCRL5 levels in PCs derived from patients with MM compared with those of healthy donors (Fig. 1a; clinical information are available in Supplementary Table S1a). Analysis of The Cancer Genome Atlas (TCGA) and GSE39754 datasets (Supplementary Fig. S1b) corroborated this result. For example, for patient 27522, the expression of FCRL5 in the primary tumor, remission, and recurrence were consistent with the tumor burden, spanning six key time points (Fig. 1b; Supplementary Table S1a). GSE143317 dataset shows elevated FCRL5 expression in PCs of relapsed MM patients after CAR-T cell therapy targeting BCMA (Fig. 1c), and elevated FCRL5 expression was associated with shorter disease-free survival times (Fig. 1d, Supplementary Fig. S1c). Quantitative analyses confirmed increased FCRL5 expression in patients with MM at our center, particularly those with chromosome 1q21 gain (Fig. 1e–g, clinical feature are available in Supplementary Table S1b). Among six MM cell lines evaluated (U266, NCI-H929, MM1.S, KMS11, ARD and RPMI-8226), NCI-H929 cells showed the highest FCRL5 expression, although the level was lower than that observed in patients with MM (Fig. 1h). Findings above emphasize the potential of FCRL5 as a promising CAR-T targeting immunotherapeutic approach.

### FCRL5 CAR-T cells bind and lyse MM cells

**Fig. 2:**
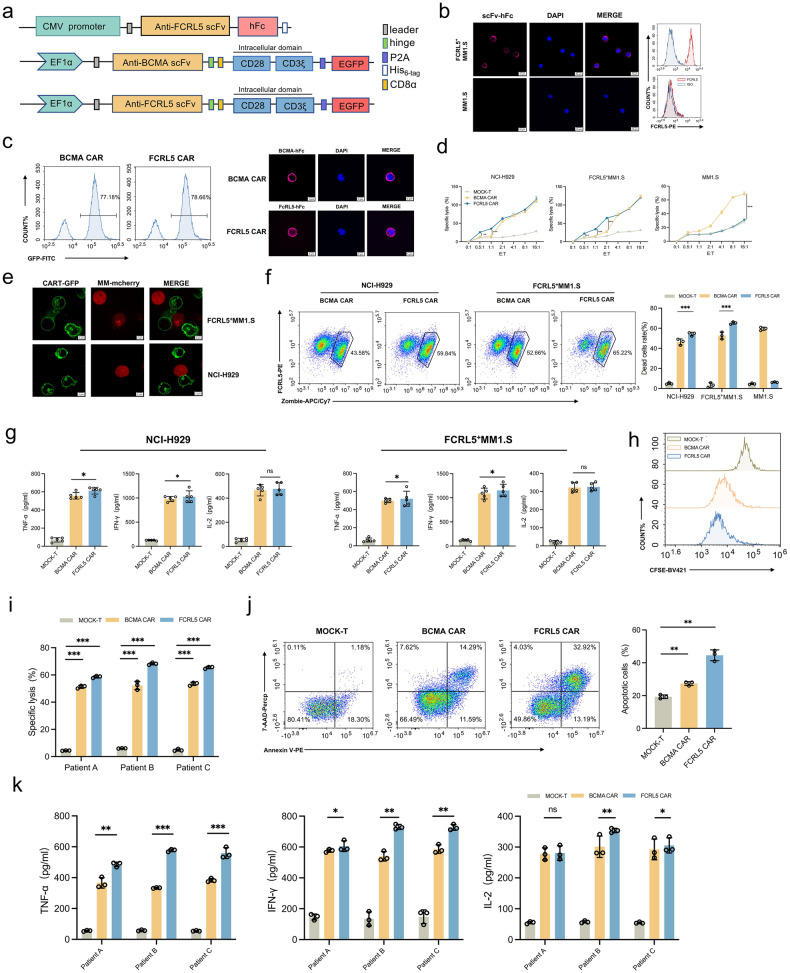
Targeting of FCRL5^+^ multiple myeloma (MM) cells by CAR-T cells**. a** Schematic illustration of FCRL5 scFv-hFc and FCRL5 CAR-T cell structures. **b** Validation of FCRL5 scFv-hFc as a primary antibody against FCRL5^+^MM1.S; MM1.S cells served as a control. Scale bar: 10 μm. **c** BCMA and FCRL5 CAR expression post-transfection analyzed using flow cytometry and immunofluorescence. Scale bar: 5 μm. **d** Cytotoxic capabilities of FCRL5 and BCMA CAR-T cells against NCI-H929 and FcRL5^+^MM1.S cells evaluated at various effector-to-target (E:T) ratios following a 24-h incubation. **P < 0.01; ***P < 0.001 compared with BCMA CAR-T. **e** Recognition capabilities of FCRL5 CAR-T cells for NCI-H929 and FcRL5^+^MM1.S cells. Scale bar^:^ 5 μm. **f** Ratio of zombie ^+^ dead cells ascertained using flow cytometry, indicating a statistically significant increase in cell death compared to Mock T cells (*** *P* < 0.001 vs. Mock T). **g** Levels of cytokines IL-2, IFN-γ, and TNF-α in the culture medium quantified using enzyme-linked immunosorbent assay (ELISA). * *P* < 0.05 compared with BCMA CAR; ns indicates no statistically significant difference. **h** CFSE dilution analyzed in co-cultured Mock T, BCMA CAR-T, and FCRL5 CAR-T cells. The analysis was specifically gated on CD8^+^ CAR-T cells at an E:T ratio of 4:1 using bone marrow mononuclear cells (BMMCs) from patients with MM with 1q21 gain (n = 3). **i**, **j** Cytotoxicity and apoptosis rates in cells from patients with MM evaluated over a 24-h period. **k** Cytokine levels in the culture medium quantified using ELISA. The results are presented as the mean ± standard deviation (*n* = 3). Statistical analysis is based on one-way analysis of variance (* *P* < 0.05; ** *P* < 0.01; ***P < 0.001 compared with BCMA CAR-T; ns indicates no statistically significant difference)

To validate the binding affinity of FCRL5 for MM cells, we conjugated an FCRL5-specific single-chain variable fragment (scFv; patent US10913796B2) with human fragment crystallizable Fc domain (hFc) to generate an FCRL5–hFC construct (Fig. 2a), which was subsequently expressed in HEK-293T cells. Among the MM cell lines the MM1.S cell line had the lowest FCRL5 expression level; therefore, we generated FCRL5-overexpression lines from MM1.S cells (FcRL5^+^MM1.S cells) to mimic the elevated FCRL5 levels in patients with MM (Fig. 1i). FCRL5–hFc exhibited selective affinity for FCRL5^+^MM1.S, but not the parental MM1.S cells (Fig. 2b). We engineered BCMA- and FCRL5-CAR (Fig. 2a) and achieved transduction rates of 77.18% and 78.66%, respectively, in primary human T cells (Fig. 2c). FCRL5 CAR-T cells exhibited a higher CD8^+^ T-cell ratio and elevated CD28 expression post transfection, whereas the T-cell phenotype ratios and CD25 and CD69 levels were comparable with those of Mock-transfected T cells (Supplementary Fig. S2a–c). In co-culture, FCRL5 CAR-T cells displayed enhanced tumor-killing ability at lower effector:target (E:T) ratios (Fig. 2d), superior recognition of NCI-H929 and FCRL5^+^MM1.S cells (Fig. 2e) and resulted in enhanced cell death than BCMA CAR-T cells (Fig. 2f). Furthermore, higher interferon-gamma (IFN-γ) and tumor necrosis factor-alpha (TNF-α) production was observed in co-culture with FCRL5 CAR-T cells rather than BCMA CAR-T cells (Fig. 2g). The carboxyfluorescein succinimidyl ester (CFSE) dilution assay confirmed an increase in FCRL5 CAR-T cell proliferation (Fig. 2h).

Chromosome 1q amplification is a frequent genetic aberration in MM correlated with disease progression. Moreover, FCRL5 expression is linked to a 1q21 gain in high-risk MM.^20^ Therefore, Effectiveness of FCRL5 CAR-T cells utilizing bone marrow mononuclear cells(BMMCs) in 1q21-amplified MM was evaluated from patients with this chromosomal abnormality (Supplementary Fig. S2d). Consistently, co-culture with FCRL5 CAR-T cells promoted considerable lysis (Fig. 2i, j) and increased TNF-α and IFN-γ production (Fig. 2k) of these BMMCs, suggesting their therapeutic potential for patients with MM exhibiting FCRL5 antigen expression.

### FCRL5 CAR with the CD28 co-stimulatory domain is more effective against MM

**Fig. 3:**
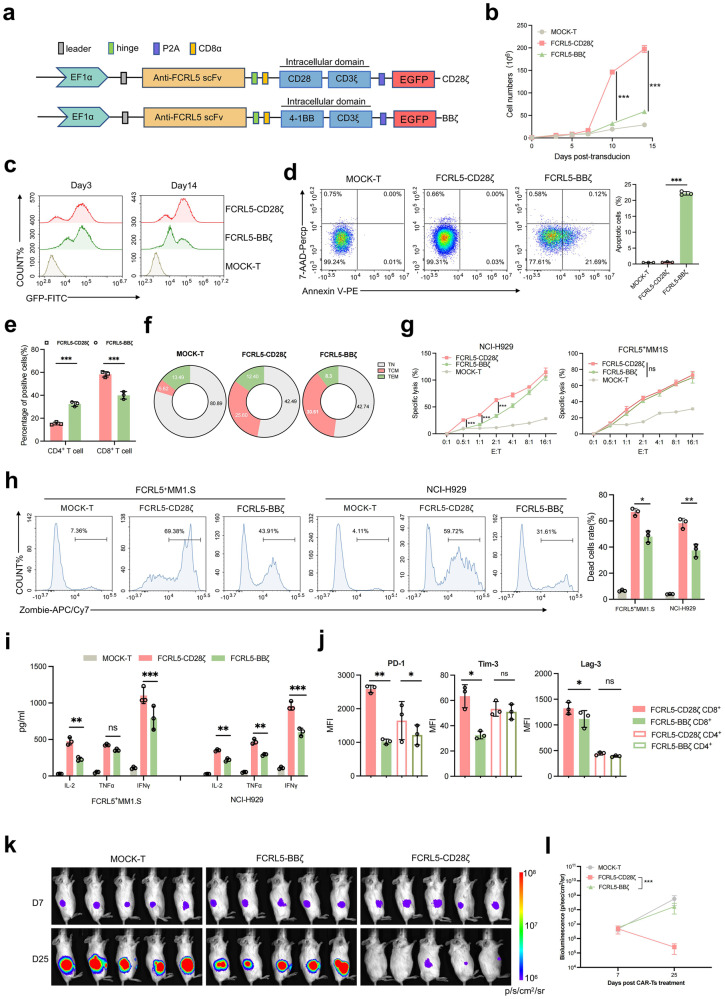
FCRL5 CARs with CD28 co-stimulatory domains are more effective against multiple myeloma (MM)**. a** Schematic diagram depicting the expression systems utilized for CAR transduction into T cells. **b** Proliferation metrics for FCRL5-specific CAR-T cells (***P < 0.001 vs. BBζ CARs CAR-T group). **c** Surface expression of CD28ζ- and BBζ-specific CARs on T cells assessed 3- and 14- days post-transduction. **d** Apoptotic rates compared between CD28ζ and BBζ CAR-T populations (***P < 0.001 vs. CD28ζ CAR-T group). **e** CD4^+^/CD8^+^ T-cell ratios in CD28^ζ^ and BBζ groups post-transduction examined via flow cytometry. **f** T-cell subtypes, classified as CD62L and CD45RO, assessed two weeks post-transduction. **g** Cytotoxicity of CAR-T cells against target cell lines evaluated using various effector:target (E:T) ratios (***P < 0.001 vs. the BBζ-CARs group; ns, no statistically significant difference). **h** Flow cytometry to quantify zombie^+^ dead cells; *P < 0.05; **P < 0.01 vs. BBζ CARs group. **i** Enzyme-linked immunosorbent assay to quantify cytokine concentrations, including IL-2, IFN-γ, and TNF-α; statistical analysis is based on one-way analysis of variance (ANOVA) and Dunnett’s test (*P < 0.05; **P < 0.01; ***P < 0.001 vs. BBζ CARs group; ns, no statistically significant difference). **j** Changes in expression of immune checkpoint markers PD-1, TIM-3, and LAG-3 in response to CD8^+^ T cells. **k** Representative bioluminescence images documenting xenograft progression after various treatments over time (*n* = 5 mice per group; *N* = 30 for all groups). Tumor flux (photons/s) was quantified using Living Image software. For the Mock T group, measurements on day +25 were excluded from the analysis due to either mortality prior to imaging or a compromised physiological state, resulting in unreliable imaging data. Statistical evaluations were conducted using repeated-measures ANOVA and log-rank tests. Data in **k** and **l** illustrate three independent experiments, each employing T cells from three different donors (*n* = 5 mice per experimental group)

To compare the efficacy of the 4-1BB and CD28 co-stimulatory domains in targeting CAR-T cells to MM, we engineered second-generation FCRL5 CARs featuring CD8α stalks and either the 4-1BB (BBζ) or CD28 (28ζ) domain (Fig. 3a). CD28ζ CAR-T cells outperform BBζ CAR-T cells with 4-fold proliferation and sustained transgene expression (Fig. 3b, c; Supplementary Fig. S3a). Fourteen days post-transduction, CD28ζ CAR T-cells exhibited diminished apoptosis, elevated CD8^+^ T cells ratio, and less memory T cells (Fig. 3d–f). Although both constructs were potent against high-antigen-density cells, CD28ζ showed superior efficacy against low FCRL5-expressing NCI-H929 cells at lower E:T ratios (Fig. 3g). In co-culture, CD28ζ CAR-T cells demonstrated enhanced cytokine secretion and tumor-killing ability than BBζ CAR-T cells (Fig. 3h, i), particularly against NCI-H929 cells with low antigen density. T-cell exhaustion markers (TIM-3, LAG-3, PD-1) were overexpressed in CD8^+^ CD28ζ CAR-T cells (Fig. 3j). In a mouse model injected NCI-H929 cells (NCI-H929-luc), treatment with CD28ζ CAR-T cells remarkably promoted tumor death and extended mice model survival, whereas BBζ CAR-T cells had minimal impact (Fig. 3k, l; Supplementary Fig. S3b). Hence, FCRL5-28ζ CAR-T cells are more effective against MM, especially in cells with a low antigen density.

### FCRL5 CAR-T cells repress established tumors in vivo

**Fig. 4:**
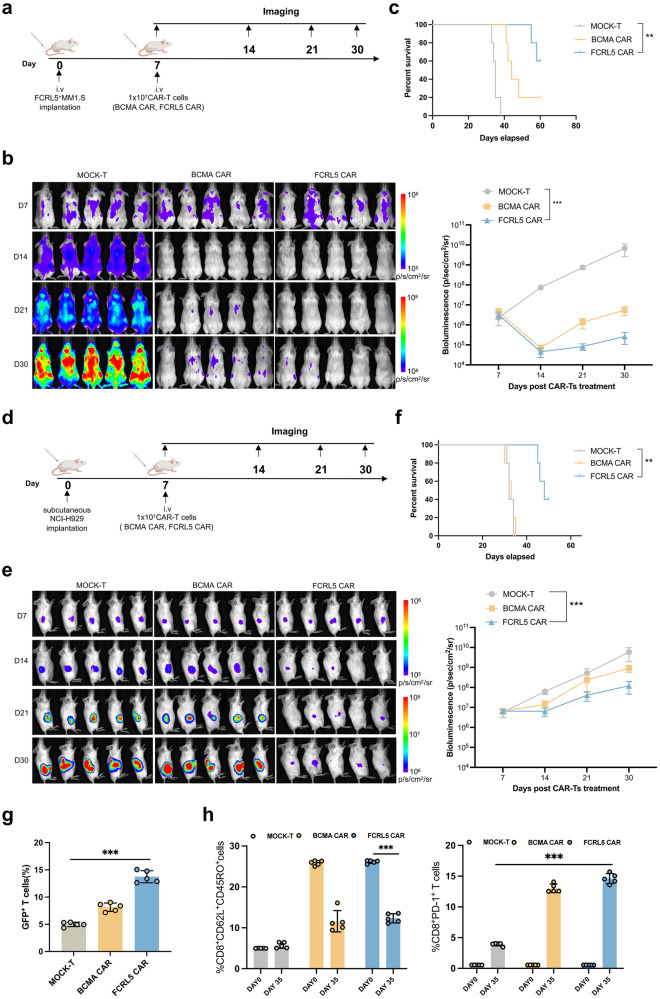
Antitumor effects of FCRL5-redirected CAR-T cells in cell line-derived xenograft models. **a** Schematic illustration of the treatment protocol for NSG mouse models with FCRL5-specific engineered T cells. **b** Representative bioluminescent images illustrating xenograft progression following different treatments over time (*n* = 5 mice per group; *N* = 60 for all groups). Tumor flux (photons/s) was quantified using the Living Image software (right panel). **c** Comparative analysis of overall survival rates in tumor-bearing mice using the log-rank statistical test (*P < 0.05; **P < 0.01; ***P < 0.001 vs. Mock T group) **d** Schematic representation of the protocol for administering FCRL5-engineered T cells in NSG mouse models. **e** Representative bioluminescence images documenting xenograft progression after various treatments over time (n = 5 mice per group; N = 60 for all groups). Tumor flux (photons/s) was quantified using the Living Image software (right panel). **f** Comparative analysis of overall survival rates for tumor-affected mice, determined via the log-rank test (*P < 0.05; **P < 0.01; ***P < 0.001 relative to the Mock T group). **g** Quantitative assessment of CAR-T-positive cellular proportions within tumor nodules (*P < 0.05, **P < 0.01, ***P < 0.001 relative to the Mock T group). **h** Rate of tumor-infiltrating CAR-T cells, focusing on central memory T cells (TCM) and PD-1-expressing CD8^+^ T cells, presented as mean ± standard deviation (*P < 0.05; **P < 0.01; ***P < 0.001 relative to baseline [day 0] or Mock T group)

Due to the difficulties in establishing consistently disseminated tumors in mice, we evaluated the in vivo antitumor efficacy of FCRL5 CAR-T cells in a disseminated MM xenograft model replacing in vitro-expanded NCI-H929 cells injection intravenously. Intravenously inoculating luciferase-expressing FCRL5^+^MM1.S(FCRL5^+^MM.1S-luc) cells into NSG mice was used to obtain model above (Fig. 4a), with CAR-T cells originating from three different donors. FCRL5 CAR-T cells suppressed the growth of FCRL5^+^MM1.S-luc xenografts, thereby extending the lifespan of tumor-bearing mice (Fig. 4b, c). In order to evaluate the tumor-killing activity of FCRL5-redirected CAR-T cells in vivo, a subcutaneous model was established by injecting 2 × 10^6^ NCI-H929-luc cells. Dose of 1 ×10^7^ CAR T-cells was administered to each mouse (Fig. 4d). The animals subjected to control Mock T-cell therapy exhibited rapid neoplastic proliferation, whereas FCRL5 CAR-T cells effectively suppressed tumor growth for 14 days. However, the tumor suppression mediated by FCRL5 CAR-T cells was temporary. Mice relapsed within 28 days and culminated in the mortality of all tumor-bearing mice by day 60 (Figs. 4e, f). Notably, tumor-infiltrating CAR-T cells showed a decrease in the proportion of TCM cells and upregulation of PD-1 expression post-treatment in the FCRL5 CAR-T cell group (Fig. 4g, h).

### FCRL5 CAR/IL-15 enhances the antitumor efficacy of CAR-modified T cells in vitro

**Fig. 5:**
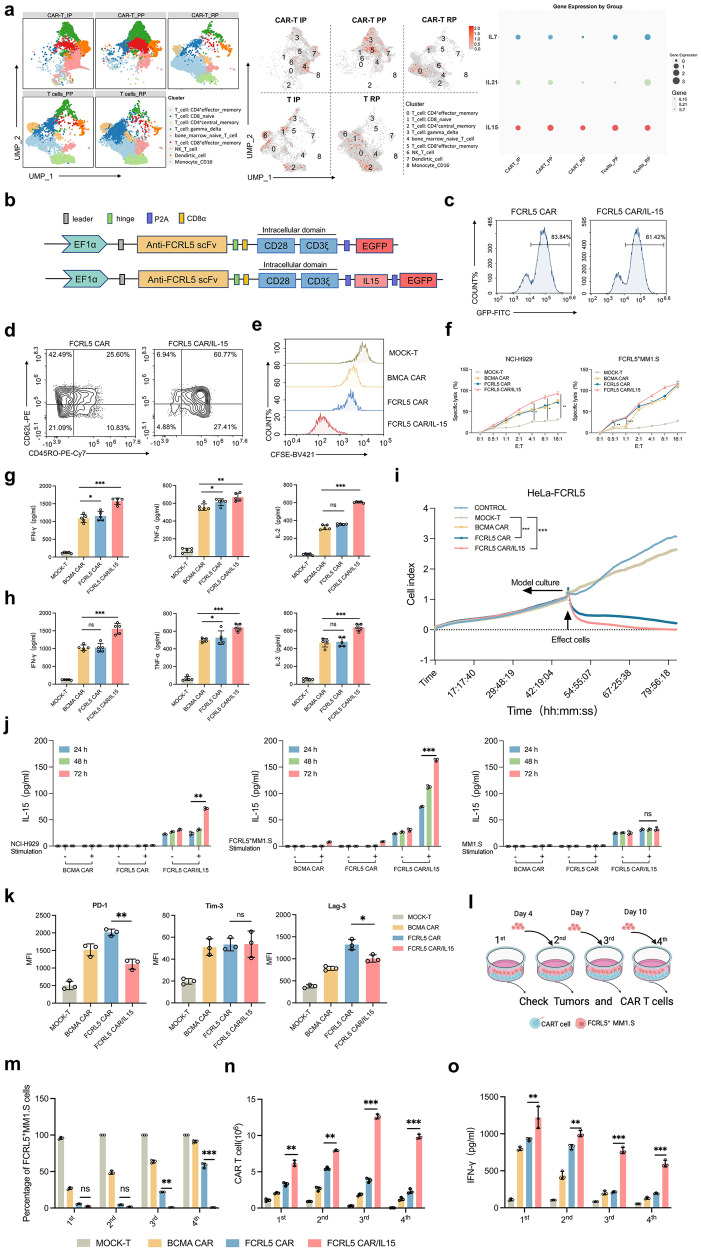
Construction of FCRL5-specific CAR-T cells with IL-15. **a** UMAP visualization of single cells collected at five discrete stages from a patient with plasma cell leukemia treated with BCMA-CAR-T cells. Cells are color-coded according to type (CD4^+^effector memory T cells, CD8 naïve T cells, CD4^+^central memory T cells, gamma delta T cells, bone marrow naïve T cells, CD8^+^ effector memory T cells, natural killer T cells, dendritic cells, CD16^-^monocytes) and IL-15 expression levels are represented by color intensities. The scatter plot encapsulates the dynamic alterations in IL-15, IL-7, and IL-21 expression across distinct stages of disease progression. Each node within the plot signifies a unique cluster within a particular disease stage, the magnitude of which is indicated by the node size. The disease stages are represented on the x-axis, whereas the y-axis portrays the unique clusters discerned within the dataset (CAR-T IP: CAR-T products before infusion; CAR-T PP: CAR-T at the peak phase on day 8 after infusion; CAR-T RP: CAR-T at the remission phase on day 15 after infusion; PP: endogenous T cells at the peak phase on day 8 after CAR-T infusion; T cell RP: endogenous T cells at the remission phase on day 15 after CAR-T infusion). **b** Schematic illustration of FCRL5-specific CAR-T cells engineered to express IL-15. **c** After transfection on day 3, CAR and CAR/IL-15 expression levels in T cells were evaluated using flow cytometry. **d** Seven days post-transduction, effector memory (TEM) and central memory (TCM) phenotypes within CD8^+^ T cells were characterized using flow cytometry using CD62L and CD45RO as identifying markers. **e** CFSE dilution reflecting cell division; the plots were gated on CD8^+^ CAR-T cells. **f** Cytotoxic effects of the CAR-T/IL-15 construct against specific cell lines at varying effector:target (E:T) ratios following a 24-h incubation period (*P < 0.05; **P < 0.01; ***P < 0.001 vs. Mock T group). **g**, **h** Concentrations of IL-2, IFN-γ, and TNF-α in the culture medium determined using enzyme-linked immunosorbent assay using FcRL5^+^MM1.S (g) or NCI-H929 (h) as the target cells (*P < 0.05; **P < 0.01; ***P < 0.001 vs. BCMA CAR group; ns, no statistically significant difference). **i** FCRL5 CAR in conjunction with FCRL5 CAR/IL-15-engineered T cells effectively targeted and lysed HeLa-FCRL5 cells. **j** Evaluation of IL-15 secretion under antigenic stimulation: T cells expressing either BCMA-CAR, FCRL5 CAR, or the FCRL5 CAR/IL-15 construct were co-cultured with NCI-H929 and FcRL5^+^MM1.S cell lines (**P < 0.01; ***P < 0.001 vs. the co-culture 24^-^h CAR group; ns, no statistically significant difference). **k** Flow cytometry assays to quantify the expression levels of the immune checkpoints PD-1, TIM-3, and LAG-3 in CD8^+^ CAR-T cells. These independent experiments were executed in triplicate with three samples per experimental group (*P < 0.05; **P < 0.01; vs. FCRL5 CAR group; ns, no statistically significant difference). **l** Schematic illustration of the protocol for sequential multi-round co-culture assays. Tumor cells were plated in 6-well culture plates 1 day prior to the introduction of T cells. On the first day (day 0), CAR-T cells were added at a 1:4 ratio to 1 × 10^6^ tumor cells, amounting to 4 × 10^6^ CAR-T cells. Subsequent assessments were conducted on days 4, 7, and 10, and 1 × 10^6^ tumor cells were added to the co-culture. **m** tumor cells; **n** CAR-T cells; **o** quantification of cytokine levels at each cycle (**P < 0.01; ***P < 0.001 compared with the FCRL5 CAR group; ns, no statistically significant difference)

To identify an appropriate target for genomic engineering of FCRL5 CAR-T cells to improve their therapeutic persistence, we evaluated the phenotypic characteristics of CAR-T and endogenous T cells in PCs from leukemia patients who received BCMA CAR-T cell therapy using the GSE151310 single-cell transcriptomic dataset. We observed a shift in CAR-T cells from a naïve T-cell state to effector-memory T-cell (TEM) and TCM phenotypes during the peak phase. Notably, IL-15 was conspicuously enriched in the CD8^+^ TEM subpopulation within CAR-T cells and in the CD4^+^ TCM cluster of endogenous T cells. Subsequent evaluation of cytokine profiles during the remission phase following CAR-T infusion indicated that IL-15 expression levels were elevated compared with those of IL-7 and IL-21 (Fig. 5a). A previous study revealed that incorporating IL-15 into the CAR-T cell culture medium more effectively enhances the antitumor activity and self-renewal.^25^ Based on these findings, we engineered CAR-T cells for autonomously secrete IL-15, achieving a robust transduction efficacy of 81.42% in primary human T cells (Fig. 5b, c). On day 7 post-transduction, FCRL5 CAR-T/IL-15 was observed to significantly elevate the levels of memory and CD8^+^ T cells, consequently enhancing the overall proliferative capacity (Fig. 5d, e; Supplementary Fig. S4a, b). For antigenic stimulation, BCMA CAR, FCRL5 CAR, and FCRL5 CAR/IL-15 T cells were cultured with NCI-H929 or FCRL5^+^MM1.S cells. FCRL5 CAR-T/IL-15 cells demonstrated markedly enhanced cytotoxic efficacy against FCRL5^+^MM1.S cells, especially when E:T ratios were 0.5:1 and 1:1, and against NCI-H929 cells at ratios above 4:1 (Fig. 5f, Supplementary Fig. S4c). These cells also manifested heightened expression of IL-2, TNF-α, and IFN-γ (Fig. 5g, h). FCRL5 CAR-T/IL-15 exhibited enhanced activation along with elevated cytotoxic markers, as evidenced by increased CD69^+^ and CD107a^+^ ratios in CD8^+^ CAR-T cells (Supplementary Fig. S4d, e), and reduced the ratios of PD-1^+^ (Supplementary Fig. S4f).

Moreover, real-time cellular analysis (RTCA) corroborated the tumor-lytic potential of FCRL5 CAR-T cells in engineered FCRL5-overexpressing HeLa cells (HeLa-FCRL5) (Fig. 5i, Supplementary Fig. S4g). IL-15 levels were evaluated in the co-culture of BCMA CAR, FCRL5 CAR, and FCRL5 CAR/IL-15 T cells respective with antigenic conditions (NCI-H929 and FcRL5^+^MM1.S cells). IL-15 concentrations were undetectable in both the antigen-stimulated and unstimulated BCMA CAR or FCRL5 CAR groups, whereas antigen stimulation led to marked IL-15 upregulation in the FCRL5 CAR-T cell/IL-15 group. In the absence of antigens, both FCRL5 CAR-IL-15 cells, whether stimulated or unstimulated, secreted low IL-15 levels (Fig. 5j). Moreover, these cells exhibited downregulation of exhaustion markers levels (Fig. 5k). Serial co-culture experiments involving FCRL5^+^MM1.S cells corroborated these results (Fig. 5l). FCRL5 CAR/IL-15 T cells uniquely sustained target cell eradication throughout four consecutive rounds of cell co-culture (Fig. 5m), with the largest numbers of CAR-T cells observed in the FCRL5 CAR/IL-15 group (Fig. 5n) and the greatest release of IL-2 and IFN-γ (Fig. 5o, Supplementary Fig. S4h). Therefore, extended co-culture demonstrated that FCRL5 CAR/IL-15 cells could achieve sustained eradication of MM. The synergistic approach also effectively targeted CD138^+^ MM patient cells with a 1q21 gain (Supplementary Fig. S4i). These findings substantiate the potent effect of IL-15 secretion on enhancing CAR-T cell-mediated anti-MM efficacy.

### Transgenic IL-15 enhances FCRL5 CAR-T cell efficacy and persistence in vivo

**Fig. 6:**
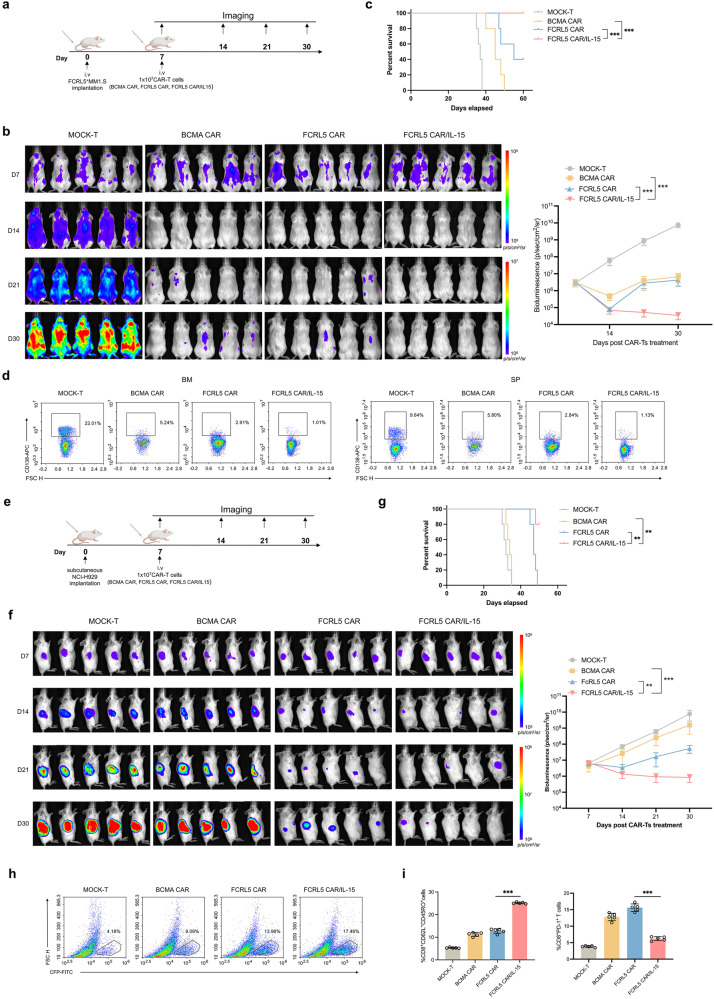
Antitumor effects of FCRL5-redirected CAR-T/IL-15 cells in cell-derived xenograft models. **a** Schematic illustration of the therapeutic protocol for NSG mouse models using FCRL5-targeted CAR-T cells. **b** Left panel: Representative bioluminescent images of xenograft models receiving assorted treatments across temporal intervals, with each group comprising five replicates (*N* = 80 NSG mice). Right panel: Quantification of aggregate tumor flux, expressed in photons per second (p/s), conducted using Living Image software. **c** Kaplan–Meier curves depict survival in tumor-bearing mice analyzed using the log-rank test (*P < 0.05; **P < 0.01; ***P < 0.001 vs. BCMA CARs group). **d** Tumors harvested on day 35 analyzed using flow cytometry for CD138 expression. Dot plots represent one randomly selected specimen per group. **e** Diagram illustrating the therap**e**utic regimen for NSG murine models utilizing FCRL5-engineered CAR T cells. **f** Left: Representative bioluminescent images of xenografts from diverse treatment groups over time (left, *n* = 5 per group; total NSG mice, N = 80). Tumor flux (p/s) was quantified using the Living Image software (right panel). **g** Log-rank test of Kaplan–Meier survival curves for tumor-bearing mice (*P < 0.05; **P < 0.01; ***P < 0.001 vs. BCMA CARs group). **h** Quantitative flow cytometric examination of the CAR-T^+^ cell proportion in tumor nodules. **i** Rate of TCM and PD-1 expression in tumor-infiltrating CD8^+^ CAR-T cells. (*P < 0.05; **P < 0.01; ***P < 0.001 vs. FCRL5 CARs group)

We further evaluated FCRL5 CAR-T/IL-15’s effectiveness against tumors using a xenograft model (Fig. 6a). FCRL5 CAR-T/IL-15 effectively inhibited FCRL5^+^MM.1S-luc tumor growth, thereby extending the survival of tumor-bearing mice (Fig. 6b, c). Flow cytometric analysis on day 35 revealed a reduced tumor burden within the spleen and bone marrow following FCRL5 CAR/IL-15 treatment (Fig. 6d), accompanied by an increase in TNF-α, IFN-γ, and IL-15 detected on days 28 and 35 (Supplementary Fig. S5a). FCRL5 CAR-T/IL-15 treatment also induces an increase in body weight starting on day 14 (Supplementary Fig. S5b). In the NCI-H929-luc subcutaneous model (Fig. 6e), both FCRL5-targeted CAR-T cell treatments initially attenuated tumor progression; however, FCRL5 CAR-T/IL-15 demonstrated enhanced therapeutic sustainability, with mice experiencing prolonged survival until the conclusion of the study (Fig. 6f, g).

The FCRL5 CAR-T/IL-15 group showed increased infiltration of CAR-T cells and a shift toward a TCM phenotype within the tumors, coupled with diminished PD-1 expression (Fig. 6h, i). Similar results were observed in the heterograft model, confirming the role of IL-15 in augmenting the antitumor activity of FCRL5-targeting CAR-T cells (Supplementary Fig. S5c–e).

## Discussion

CAR-T therapy is a cutting-edge modality in oncological immunotherapy^29,30^ and has been recognized as a potential therapeutic avenue for MM.^31,32^ BCMA is a target for MM CAR-T therapy due to its predominant expression on malignant cells. Several clinical studies have demonstrated favorable outcomes by treating MM using BCMA CAR-T therapy. Notably, one study focusing on elderly patients, including those over 70-years-old, demonstrated favorable efficacy and safety outcomes, with a notable achievement of an 82% objective response rate (ORR) and a segment of 40% patients achieving a status of very good partial response (VGPR) or higher.^33^ GPRC5D, highly expressed on myeloma cells, exhibited 71-100% remission rates in several Phase I studies of GPRC5D CAR-T cells for MM treatment.^16^ The CS1 CAR-T cells display anti-MM activity in vitro and in murine xenograft models. Similarly, refractory and relapse of patients with MM treated with CS1-BCMA CAR-T exhibited an 81% ORR, with 6 cases achieving stringent complete response (sCR).^34^ Moreover, diversified manufacturing strategies, such as incorporating IL-2, enriching memory-like phenotype T-cells, and employing dual targeting, enhance CAR-T therapy outcomes.^35,36^ Although patients with MM treated with CAR-T therapy exhibit a high primary response rate, the early high relapse rate remains a challenge. Consequently, we analyzed single-cell sequencing datasets derived from patients with MM, which revealed a considerable downregulation in BCMA expression in tumor specimens from individuals with relapsed MM following BCMA-targeted CAR-T cell treatment, as shown in Fig. 1c. Consistently, patients with loss of or low antigen expression have shown a diminished therapeutic response to CAR-T cell intervention.^12,37^ Given the limitations of current target antigens, it is imperative to explore alternative molecular targets to enhance the efficacy and safety of CAR-T strategies for MM therapy.

FCRL5, a member of the immunoglobulin superfamily, is associated with B-cell ontogeny and lymphomagenesis,^21^ making it a promising target for antitumor therapies.^20,38^ Our serial analysis of FCRL5 expression in patients with MM, from primary tumors to relapse, substantiated its stable presence as a therapeutic target. Notably, the observation of elevated FCRL5 levels in cases of recurrent MM after CAR-T cells targeting BCMA treatment underscores its potential as a target for therapeutic adaptability. Our data demonstrated the upregulation of FCRL5 mRNA and protein expression in bone marrow specimens derived from MM patients. In vitro assays and in murine xenograft models revealed that FCRL5-CAR-T cells displayed enhanced cytotoxicity against NCI-H929 and FCRL5-overexpressing MM1.S cell lines compared with BCMA CAR-T cells, especially at lower effector: target ratios.

To enhance the effects of CAR-T cells against MM, a second-generation FCRL5 CAR was engineered to feature either the CD28ζ or 4-1BBζ as the co-stimulatory domain. Results showed that CD28ζ CAR-T cells exhibited high proliferative capacity and sustained transgene expression. CAR-T cells with CD28ζ as the co-stimulatory domain displayed superior tumor lysis and cytokine secretion against low-antigen-density MM cells in vitro and demonstrated markedly enhanced antitumor activity and survival benefits in vivo using xenograft mouse models compared to 4-1BBζ CAR-T cells. Similarly, 4-1BBζ signaling in CARs has been shown to trigger T cell apoptosis, thereby hampering their antitumor activity.^39^ Further investigations assert that CD19-CD28ζ showcases better efficacy compared with CD19-4-1BBζ in counteracting low antigen tumors.^40^ Nevertheless, only partial tumor remission was achieved with FCRL5-CD28ζ CAR-T-cell administration. In refractory tumor models, our results showed diminished CAR-T-cell tumor infiltration, elevated expression of immunosuppressive markers, and a reduced number of TCM cells, suggesting that intrinsic adaptive mechanisms within the tumor may regulate T-cell phenotypes, thereby constraining their antitumor efficacy.^12,37,41^ Improving CAR-T cell persistence enhance antitumor effectiveness both in co-culture of cells and mouse models.^42,43^ To address tumor-induced CAR-T cell persistence deficiencies, we observed a dynamic transition in CAR-T phenotypes from naïve to both TEM and TCM states at their therapeutic peak, with a notable enrichment of IL-15 in the CD8^+^ TEM and CD4^+^ TCM subsets and subsequent overrepresentation of IL-15 compared with IL-21 and IL-7 during the remission phase. IL-15 has the potential to boost CAR-T- cell antitumor activity and self-renewal capability by enhancing cytotoxicity and balancing the tumor microenvironment.^25^ This suggests a strategy to enhance the effectiveness and durability of CAR-T cell immunotherapy. Through the modification of the CAR-T-cell structure for IL-15 secretion, we were able to elevate the ratio of TCM cells and improved antitumor efficacy in the refined FCRL5 CAR-T/IL-15 model, which showed increased intertumoral CAR^+^ T-cell infiltration, reduced PD-1 expression, and potent tumor-suppressive effects in xenografts by days 28 and 35 post-treatment, validating the viability of this construct for targeted MM treatment. Given the potential “stemness” property conferred by IL-15,^25^ our study revealed that the number of IL-15-augmented CAR T cells diminished following the fourth co-culture cycle, indicating a controlled proliferative capacity, as shown in Fig. 5n. Additionally, these IL-15-augmented cells led to the reduction in the ratio of cells of the TCM phenotype.

Chromosome 1q amplification is a frequent genetic abnormality observed in MM patients and serves as a biomarker of aggressive disease.^44^ Recent studies have shown that bortezomib and immunomodulatory agents do not improve adverse effects or prognosis in patients with MM harboring 1q amplification.^45^ Li et al.^20^ demonstrated that FCRL5 overexpression is associated with gain of 1q21 in high-risk MM patients. Compared with BCMA CAR-T cells, FCRL5 CAR-T cells showed improved efficacy against MM with 1q21 gain; therefore, these patients might benefit more from FCRL5 CAR-T/IL-15 treatment, offering potential advantages to those with high-risk MM and refractory recurrence.

In this study, we demonstrated consistent FCRL5 expression, even in situations where BCMA expression diminished, following the administration of BCMA-directed CAR-T cells, and, based on this finding, engineered FCRL5-specific CAR-T cells adept at recognizing FCRL5 in MM cells and impede tumor growth both in vitro and in murine xenograft models. FCRL5/IL-15 CAR-T cells promoted a shift towards the TCM phenotype while reducing PD-1 exhaustion, which may facilitate more durable and potent anti-tumor responses, thus potentially overcoming the limitations of BCMA-targeted therapy. However, considering the limitations of the mouse models utilized in our research, we intend to use more reliable PDX models for validation and explore new regulatory mechanisms to modulate CAR-T cell activity and proliferation, thereby enhancing therapeutic safety.^46,47^ Our findings propose new avenues for future MM treatment and clinical applications.

## Materials and methods

### Cell lines and model animals

Human myeloma cell lines (NCI-H929, U266, MM1.S and RPMI-8226), human embryonic kidney epithelial cell (HEK-293T) and human cervical cancer cell HeLa were acquired from the American Type Culture Collection (ATCC; USA). The human myeloma cell line KMS11 and the ARD cell line was kindly donated from Prof. Yuhuan Zheng (West China hospital, Sichuan University). The ADR, KMS11, NCI-H929, U266, MM1.S and RPMI-8226 cells were cultured in RPMI-1640 medium (Thermo Fisher Scientific). HEK-293T cells were maintained in DMEM (Gibco; USA). Cell line culture media was supplemented with 10% fetal bovine serum (Gibco; USA), penicillin and streptomycin (1.0 mmol/L, HyClone; USA). Cell lines were grown at 37 °C in a humid incubator with 5% CO_2_.

Female NSG and B-NDG mice were obtained from Nanjing University’s Model Animal Resource Information Platform and maintained in a pathogen-free setting at the State Key Laboratory of Biotherapy, Sichuan University. All the experimental procedures were performed in compliance with the Guide for the Care and Use of Laboratory Animals of Sichuan University and approved by the Animal Care and Use Committee of Sichuan University (reference numbers: 2018–061).

### Bioinformatic analysis

MM-related datasets (GSE39754, GSE223060, GSE143317, and GSE136324) by provided the GEO database. Survival time was predicted using the GSE57317 and GSE4204 datasets.^48^ Using these GEO datasets and TCGA, we examined the expression of FCRL5 between patients with new diagnoses and those with recurrent MM; the single-cell expression and distribution of FCRL5 were compared using the GSE223060 and GSE143317 datasets.^12,49^ Public single-cell RNA-seq data analysis employed Seurat for integration and UMAP reduction, identifying nine clusters through SNN graphs and PCA. Unique markers were pinpointed via FindMarkers function, followed by enrichment analysis on markers with *P* values < 0.01. Variations in FCRL5 expression across tumor and healthy tissues were investigated using the HPA dataset (proteinatlas.org). The “ggplot2” was utilized to generate the volcano plots and heatmaps.

### Total RNA isolation and RT-qPCR analysis

Bone marrow fluid (500 µL) from patients with MM and controls underwent RNA extraction and quality assessment. FCRL5 gene expression was analyzed via qRT-PCR (BlazeTaq™ SYBR® Green qPCR Mix2.0), using GAPDH as a reference. This investigation adhered to the Declaration of Helsinki and received approval from the Ethics Committee of West China Hospital at Sichuan University (reference: 2018–061). All participants provided written informed consent.

### Construction of FCRL5-hFc recombinant protein

The amino acid sequence of anti-FCRL5 scFv (comprising VL and VH) was sourced from a patent (US10913796B2) and incorporated into both the FCRL5-scFv fusion protein and corresponding CAR constructs. This antibody has been demonstrated to attach to human MM cells and is affinity-mature.^20^ The cDNAs for the FCRL5-targeted scFv were custom-synthesized by Genewiz. Subsequently, a His tag was added to the carboxy-terminus of the FCRL5-scFv recombinant cDNA to subclone it into a eukaryotic expression vector for protein purification.

HEK-293T cells were transfected with the designated expression vectors and maintained in serum-free medium (FreeStyle™; Thermo Fisher Scientific, USA) in a humid incubator at 37°C with a 5% CO2. After incubation for 5 days, Ni-NTA affinity columns were used to purify the recombinant FCRL5 scFv-hFc protein. Protein further purification step was carried out utilizing a Superdex 200 Increase column (10/300 GL) (GE Healthcare, USA). Sodium dodecyl sulfate-polyacrylamide gel electrophoresis was employed to evaluate the size and quality of the purified FCRL5 scFv protein after staining with Coomassie brilliant blue.

### Immunofluorescence

HeLa or MM cells were seeded in 24-well plates, each containing a coverslip, and were incubated for 24 hours at 37 °C. Afterward, the cells underwent fixation in a 4% paraformaldehyde-phosphate-buffered saline (PBS) mixture for a duration of 15 minutes, followed by staining utilizing purified FCRL5 coupled with hFC (FCRL5 scFv-hFc) or primary antibody. In parallel, CAR-T cells infected with lentiviruses expressing CAR for 72 h were incubated with the FCRL5-hFc or BCMA-hFc recombinant protein. The cells were then stained for 1 h with an Alexa Fluor® 647 anti-human IgG2a antibody (Abcam,UK). As illustrated in Fig. 2e, these green fluorescent CAR-T cells and mCherry-expressing tumor cells were co-cultured. Finally, 4′,6-diamidino-2-phenylindole (DAPI; Solarbio, USA) was applied to the slides for staining. Following immunostaining, images were photographed under a confocal microscope.

### Design of CAR structure

The FCRL5-directed CAR construct comprised the original CD8α antigen, CD8 transmembrane, FCRL5-specific scFv, hinge, human CD28 or 41BB cytoplasmic domain, and CD3ξ domain. The IL-15 sequence was added immediately after the CD28/CD3ξ sequence to create the IL-15 autocrine CAR. The BCMA scFv sequence, which was obtained from Bluebird Bio, was substituted with the FCRL5 scFv sequence to generate a control CAR for comparison. A shortened and inactive human epidermal growth factor receptor gene was used to detect CAR and undesired expansion control.^46,47^

### Lentiviral preparation, purification, and T-cell transduction

Lentivirus particles of all aforementioned CARs were generated in HEK-293T packaging cells by co-transfecting the target plasmid at a 4:3:2 ratio with packaging plasmids (psPAX2 and pMD.2 G) using polyethyleneimine (PEI, Sigma-Aldrich, USA). Post transfection, the supernatants were harvested at 24 and 48-hour intervals, followed by ultracentrifugation at 25,000 × *g* for 2 hours at 4 °C for concentration. Peripheral blood mononuclear cells (PBMCs) from healthy donors were separated using gradient centrifugation at 800 × *g* for 15 min to produce T cells. The collected PBMCs were isolated by density gradient centrifugation using Ficoll-Hypaque (Sigma-Aldrich, Germany), then were cultured in X-VIVO 15 medium (Lonza, Switzerland) containing 10% human AB serum (Sigma-Aldrich, Germany),100 U/mL penicillin and 100 g/mL streptomycin in a 37 °C incubator with a 5% CO_2_ environment. The T cells were activated by stimulation with 100 ng/mL anti-CD28 monoclonal antibody (BioLegend, USA), 200 ng/mL anti-CD3 monoclonal antibody (BioLegend, USA), and 100 U/mL recombinant human IL-2 (rhIL-2) (PeproTech, New Jersey). Subsequently, T cells underwent lentiviral transduction using either BCMA-CAR, FCRL5 CAR, or FCRL5 CAR/IL15 constructs. Twelve hours post-transduction, the cells were transferred to X-vivo medium cultured with 100 U/mL rhIL-2. The lentiviral titer was calculated according to the median tissue culture infectious dose. Mock T-cells, as a negative control, were generated through transduction with a CAR vector lacking a gene construct. Control T cells, or “naïve T cells,” underwent no transduction.

### T-cell Functional Analysis

At day 10 post-transduction, T cells were isolated, purified, and stained for 30 min at 4 °C with fluorochrome-conjugated monoclonal antibodies for the markers CD8, CD4, CD62L, and CD45RO in 1× PBS buffer containing 2% fetal bovine serum.

For the co-culture experiments, FCRL5^+^MM1.S, NCI-H929, and MM1.S cells were seeded in 24-well plates at a density of 1 × 10^5^ cells/well and incubated overnight. Subsequently, these cells were co-cultured with 4 × 10^5^ effector cells—including variants of BCMA CAR-T, Mock T, FCRL5 CAR-T/IL15, and FCRL5 CAR-T—for 48 h at 37 °C. To assess T-cell activation, the cells were harvested and subjected to staining protocols using anti-CD28 and anti-CD69 antibodies.

The stimulating cells were treated with mitomycin C (Selleck, USA) for 30 min and then 1 × 10^5^ mCherry-labeled FCRL5^+^MM1.S and NCI-H929 cells were incubated in 100 μL complete culture medium alongside 4 × 10^5^ green fluorescent protein (GFP)-labeled CAR-T effector cells, which were suspended in 100 μL of the same medium and marked with CFSE. Following a three-day incubation period, the cellular characteristics were analyzed using an ACEA NovoCyte flow cytometer (Agilent Biosciences, USA).

### Lactate dehydrogenase (LDH) assay

The LDH assay is a robust method for gauging the antigen-specific cytotoxic efficacy of FCRL5-targeted CAR-T cells in vitro. The various CAR-T cell variants, including Mock T, BCMA CAR-T, FCRL5 CAR-T, and FCRL5 CAR-T/IL15, were co-cultured with MM1.S and NCI-H929 cells at E:T ratios ranging from 0:1 to 16:1. LDH released into the supernatant was quantified using an LDH cytotoxicity test detection kit (Beyotime, China), strictly following the manufacturer’s guidelines.

### Real-time cytotoxicity (RTCA) assays

We employed the xCELLigence RTCA SP system (ACEA Bioscience Inc.) to assess the cytolytic capacity of different CAR-T cell variants^50^ (Mock T, BCMA CAR-T, FCRL5 CAR-T, and FCRL5 CAR-T/IL15). FCRL5-HeLa tumor cells were seeded in an E-plate 96 (ACEA Bioscience) at a density of 10^4^ cells per well and cultured for approximately 24 h before the introduction of CAR-T cells at an E:T ratio of 4:1. Data collection and analysis were performed in compliance with the manufacturer’s protocol using RTCA Software 2.1.

### Cytokine production assays

The tumor cell lines FCRL5^+^MM1.S and NCI-H929 were co-cultured with 4 × 10^5^ effector cells of varying types—FCRL5 CAR-T/IL15, FCRL5 CAR-T, BCMA CAR-T and Mock T—in 24-well plates at 37 °C in cytokine-free medium for 24 h. The culture media were assayed for the presence of the cytokines IL-2, IFN-γ, and TNF-α at 24 h of culture. IL-15 levels were monitored at 24, 48, and 72 h using enzyme-linked immunoassay kits (BioLegend, USA). Concurrently, the various effector cells were co-cultured with BMMCs obtained from patients with MM. After incubation, the cells were stained for CD138 and FcRL5 and subjected to flow cytometry.

### Flow cytometry

After dual PBS washes, 2 × 10^5^ cells were incubated with 1% bovine serum albumin buffer (with Fc blocker, BioLegend, USA) for 0.5 h. Fluorochrome-labeled antibodies against an array of markers (CD45, CD38, CD138, and CD69) were obtained from BioLegend. Annexin V/FITC and 7AAD staining kits (4A Biotech,China) were used to identified apoptotic cells, whereas dead cells were marked using the Zombie UV Fixable Viability Kit (BioLegend, USA). FCRL5-specific scFv was validated using a primary antibody against purified FCRL5 scFv-hFc and a phycoerythrin-labeled secondary anti-human IgG antibody (BioLegend, USA). GFP served as a marker for CAR-T cells, and FcRL5^+^MM1.S and NCI-H929 cells were identified using mCherry-Red labeling.

For data analysis, FlowJo 9.3.2 software was utilized to investigate T-cell phenotype and activation. The transfection efficiency and FCRL5 expression in CAR-T cells and MM cells respectively, and cell viability were evaluated by the ACEA NovoCyte system (Agilent Biosciences, USA).

### Animal models

The intravenous model was established in female NSG mice with injection of 2 × 10^6^ FCRL5^+^MM1.S-luc cells and the subcutaneous model was injected of 5 × 10^6^ HCI-H929-luc cells on the same day (day 0). As shown in Supplementary Fig. S5, B-NDG mice (7-week-old females, *n* = 5) were intravenously injected with 5 × 10^6^ PBMCs, followed by intravenous implantation of 5 × 10^6^ FCRL5^+^MM1.S-luc cells two week later. The longitudinal tumor growth was monitored by bioluminescence imaging (BLI). On day 10, BLI measurements were performed and the mice with successful tumor establishment, defined as the growth of 1–2 tumors of equal size in the bone marrow or subcutaneous area, were divided into four treatment groups randomly. Animals with no tumor formation or showed obvious signs of distress and pain were excluded.^51^ On designated days, CAR T cells collected from the cryovials were washed and prepared for intravenous injection. Mock T cells in the same quantity as the control group were used for both tail vein and subcutaneous injection. Each mouse received 1 × 10^7^ FCRL5 CAR-T/IL15, FCRL5 CAR-T, or BCMA CAR-T cells. The mice were monitored twice daily. Isoflurane anesthesia was used to minimize pain and distress, adhering to ethical guidelines for animal research. Furthermore, mice that were obviously distressed or in pain, including those that were unable to drink or feed or had a body weight reduction of more than 20%, were humanely euthanized before the end of the study, authorized by the Institutional Animal Care and Use Committee.

### Statistical analysis

Statistical evaluations were performed using GraphPad Prism 9.0 software. Data are reported as mean ± standard deviation. Continuous variables were compared between two groups using an unpaired two-sided *t-*test. The Wilcoxon signed-rank test was used for non-normally distributed datasets. One-way analysis of variance was used to compare continuous variables across multiple groups, followed by post-hoc analysis using Dunnett’s test. Survival analysis in the MM models was performed using Kaplan–Meier estimates and significance was determined by the log-rank test. The experiments incorporated three biological replicates from each donor and were conducted twice. Differences associated with *P* < 0.05 were considered statistically significant.

### Supplementary information

SUPPLEMENTAL MATERIAL

## Data Availability

The data that support the findings of this study are available from the corresponding author upon reasonable request. Some data may not be made available because of privacy or ethical restrictions.
