# Supplementary material for: Fc receptor-like 5 (FCRL5)-directed CAR-T cells exhibit antitumor activity against multiple myeloma
Source: Signal Transduct Target Ther. 2024 Jan 12;9:16. doi: 10.1038/s41392-023-01702-2 (PMC10784595; doi:10.1038/s41392-023-01702-2)
Supplement: Supplementary file 1 — SUPPLEMENTAL MATERIAL [file 41392_2023_1702_MOESM1_ESM.docx]

**Supplementary Material**

**Fc receptor-like 5 (FCRL5)-directed CAR-T cells exhibit antitumor activity against multiple myeloma**

Zhengyu Yu†^1^, Hexian Li†^1^, Qizhong Lu^1^, Zongliang Zhang^1^, Aiping Tong^1*^, Ting Niu^1*^

^1^Department of Hematology, State Key Laboratory of Biotherapy and Cancer Center, West China Hospital, Sichuan University, Chengdu, 610041, China.

Correspondence should be addressed to [aipingtong@scu.edu.cn](mailto:aipingtong@scu.edu.cn); [niuting@wchscu.cn](mailto:niuting@wchscu.cn)

†Zhengyu Yu and Hexian Li contributed equally to this work and are co-first authors.

**This file includes:**

**Figures. S1 to S5**

**Table. S1**

**
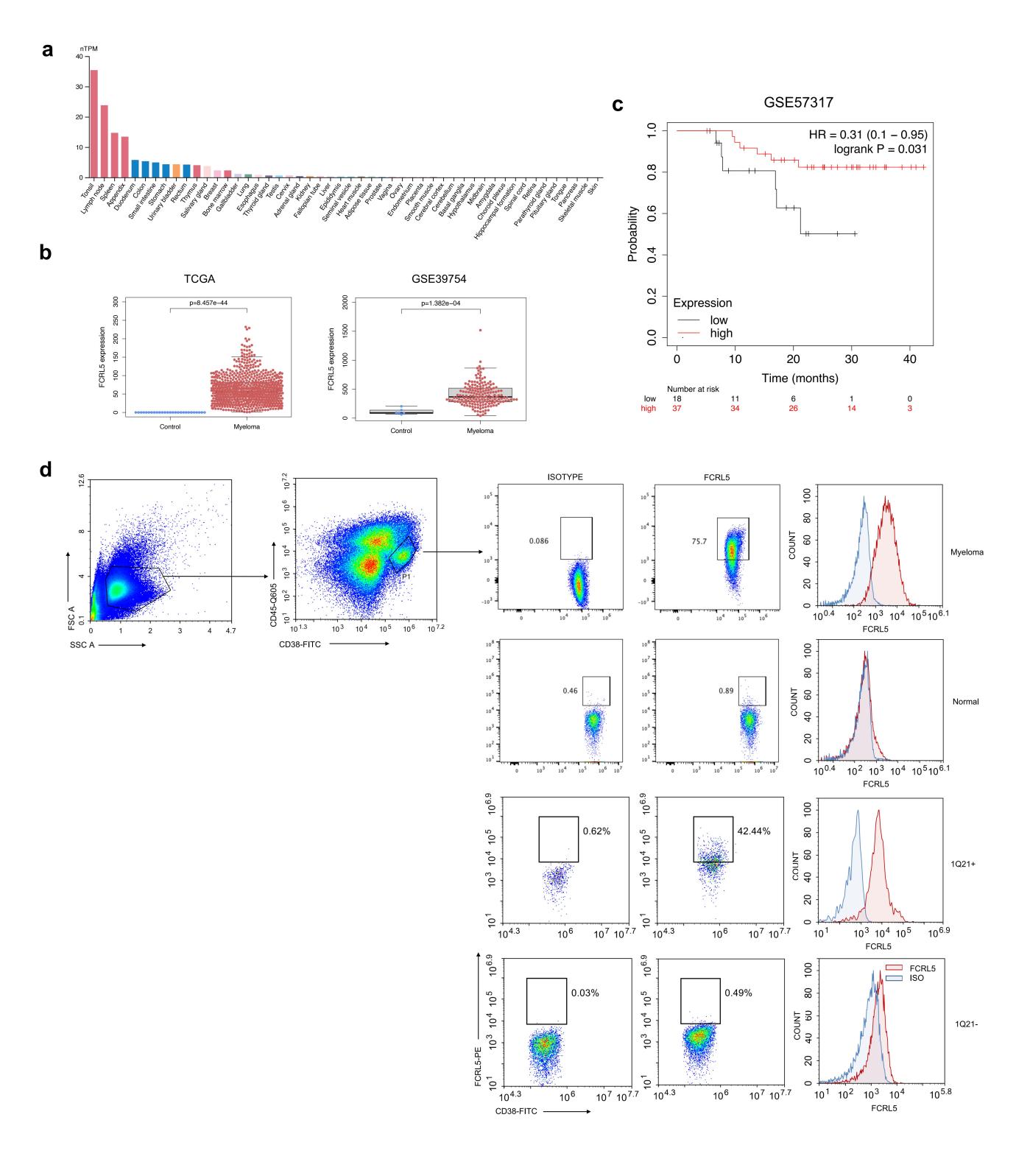
**

**Supplementary** **Fig. S1** FCRL5 expression levels in normal and multiple myeloma (MM) tissues**. a** Expression levels of *FCRL5* mRNA across different human cell types measured in terms of normalized transcripts per kilobase of exon per million reads (nTPM), as acquired from the Human Protein Atlas. The tissues were functionally categorized and coded. **b** Comparative *FCRL5* RNA-seq analysis in the bone marrow from healthy (GTEx) donors and patients with MM (TCGA); initial *FCRL5* mRNA expression in untreated MM was evaluated using the GSE39754 dataset. Data were compared with Student’s *t*-test. **c** Correlation analysis of *FCRL5* expression with disease-free survival in MM assessed using the GSE57317 dataset; groups are segmented by median module scores; significance was determined via the log-rank test. **d** Gating strategies employed for flow cytometry analyses presented in Figs. 1, 3, and 5. Cells were incubated with either anti-FCRL5-PE or IgG-PE controls. FCRL5/CD38 combinations are compared with isotype/CD38 controls, indicating the presence of CD45dim plasma cells in patients with MM and healthy controls.


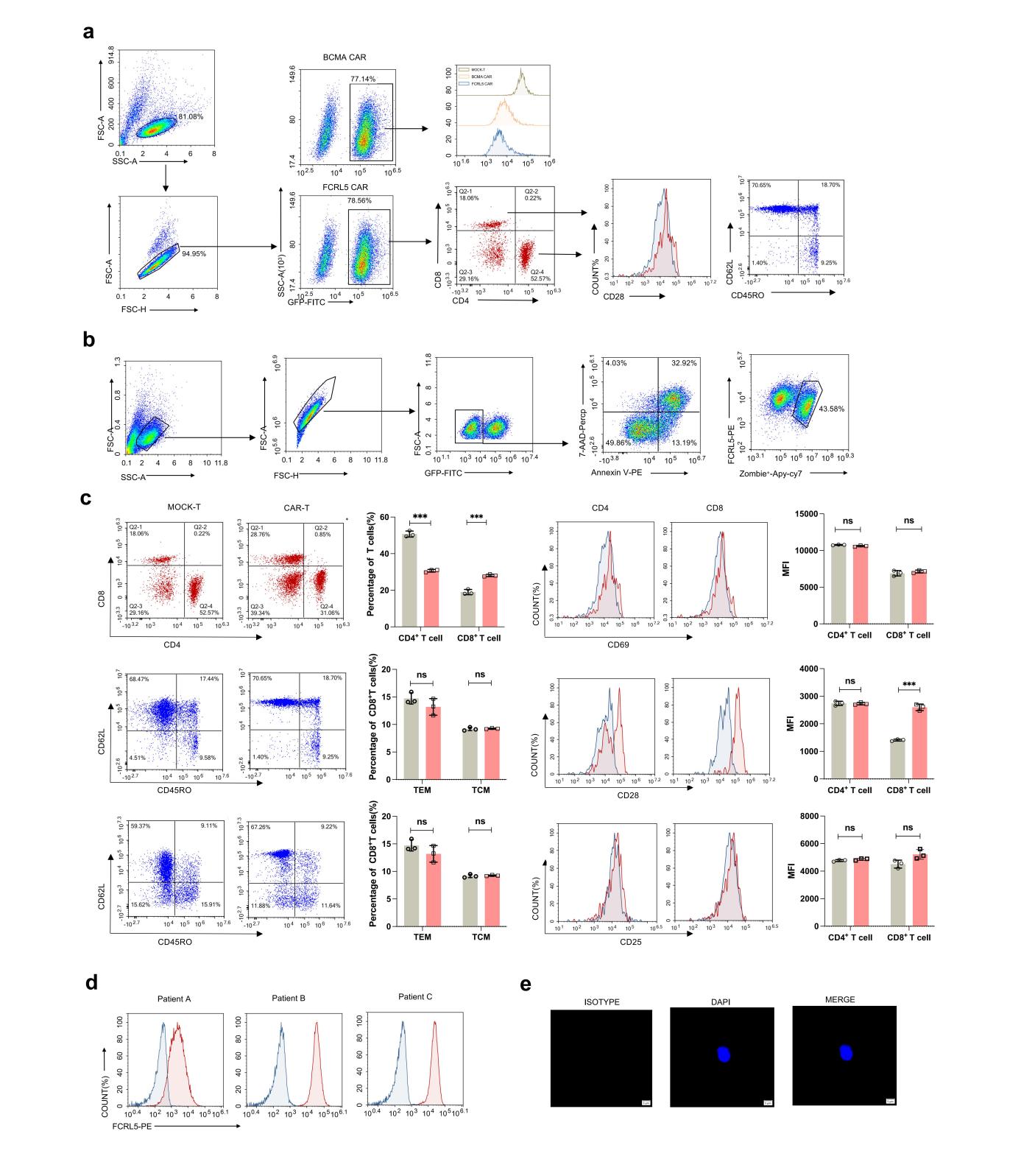


**Supplementary** **Fig. S2** Engineering and phenotypic characterization of FCRL5-targeted CAR-T cells. **a-b** Detailed gating strategies deployed for the flow cytometry analyses, which are further expounded in Figs. 2–6. **c** Post-transduction assessment of CD4^+^ and CD8^+^ T cell ratios along with key phenotypic markers (CD69, CD28, CD25, TEM, and TCM) within the responsive CD4^+^ and CD8^+^ T cell populations. **d** Assessment of FCRL5 expression in patients with multiple myeloma (MM) with 1q21 gain. **e** FCRL5 CAR expression post-transfection analyzed using immunofluorescence in the isotype. Scale bar: 5 μm. All experiments were performed independently in triplicate. The data in the R plots are specifically gated on CAR-T cells and are presented as the mean ± standard deviation. ****P* < 0.001 vs. the Mock T group; ns, no statistically significant difference.

**
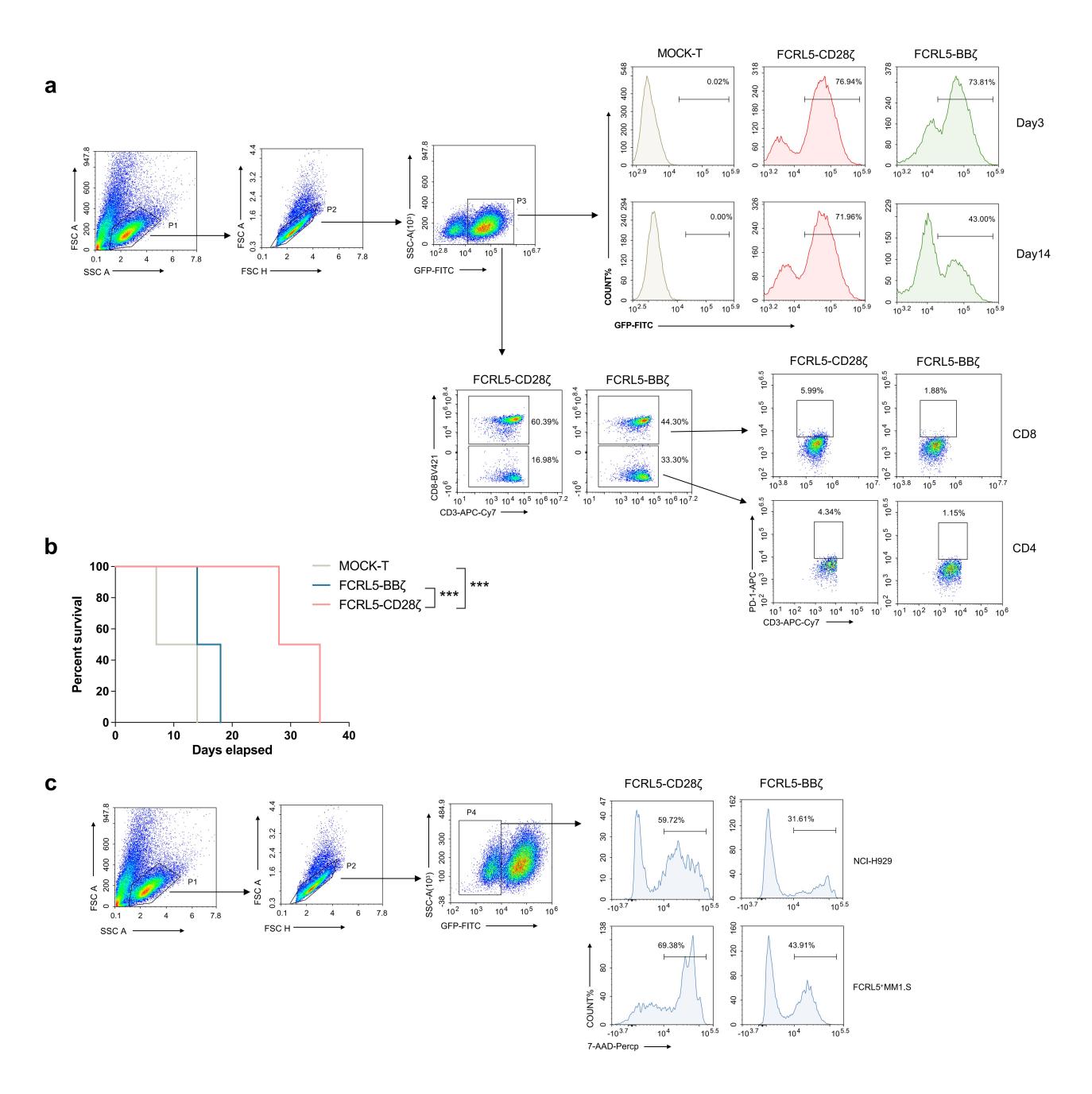
**

**Supplementary Fig. S3** Characterization of FCRL5 CAR-T cells with CD28 co-stimulatory domains in multiple myeloma. **a,c** Flow cytometry gating strategies for Figs. 3–6. **b** Survival curves for mice subjected to different treatments.

**
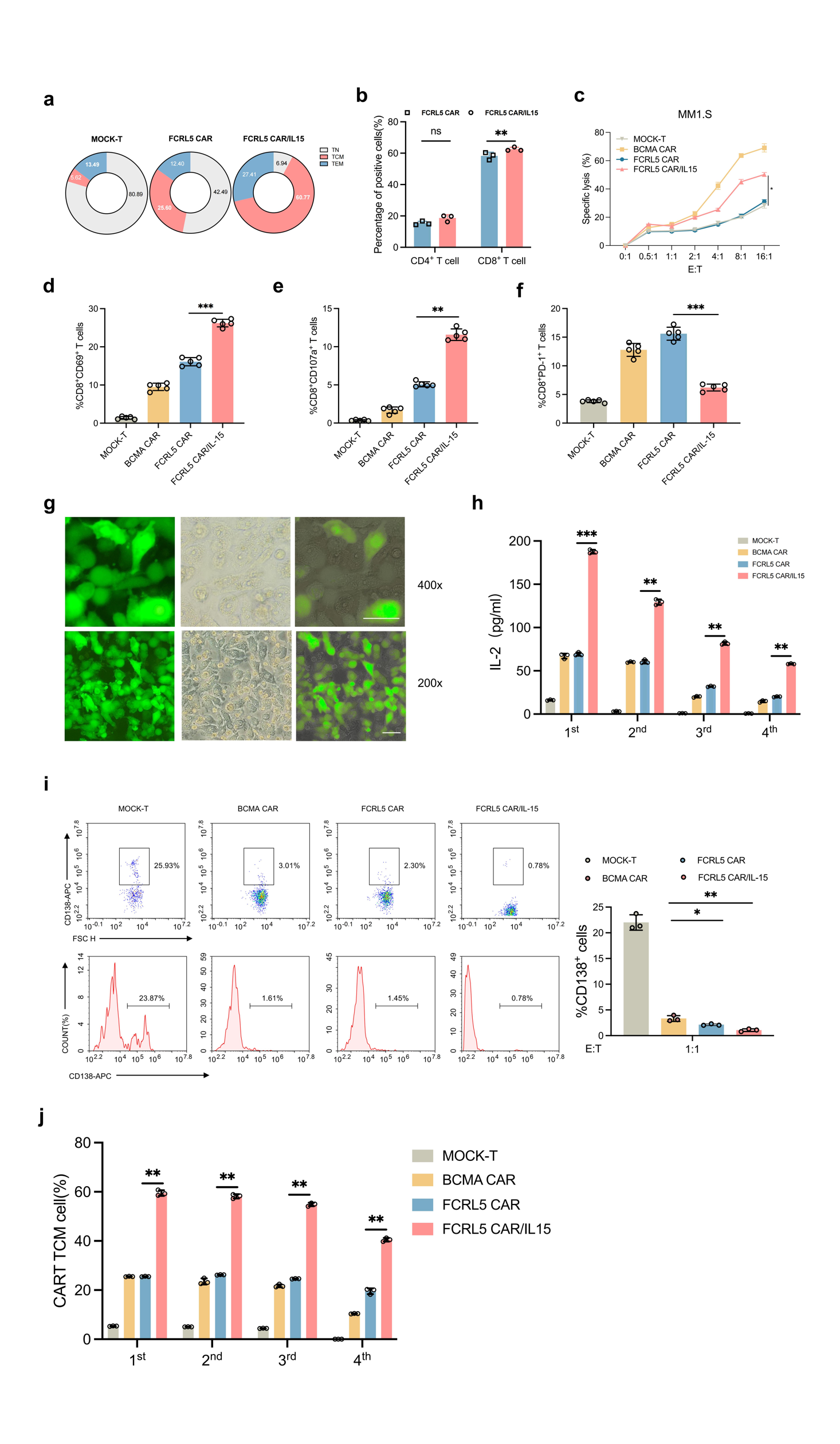
**

**Supplementary Fig. S4** FCRL5 CAR-T IL-15 has increased therapeutic efficacy against multiple myeloma (MM). **a** Relative rates of TN, TCM, and TEM CAR-T cells 7 days post-transduction. **b** CD4^+^ and CD8^+^ T cell ratios of FCRL5 CAR and FCRL5 CAR/IL-15 detected using flow cytometry. (***P* < 0.01 vs. FCRL5 CARs group; ns, no statistically significant difference). **c** Cytotoxicity of the CAR-T cell construct against MM1.S cells at different effector: target (E:T) ratios for 24-h incubation (**P* < 0.001 vs. Mock T group). **d-f** CD69, CD107a, and PD-1 ratios of the FCRL5 CAR/IL-15 group detected using flow cytometry (***P* < 0.01; ****P* < 0.001 vs. FCRL5 CARs group). **g** Immunofluorescence staining for the surface expression of FCRL5 (green) in FCRL5-overexpressing HeLa cells. Scale bars = 550 μm. **h** Concentration of IL-2 in the culture medium measured using enzyme-linked immunosorbent assay (***P* < 0.01; ****P* < 0.001 vs. FCRL5 CARs group). **i** Co-culture of FCRL5 CAR-T cells or their matched Mock T cells with bone marrow mononuclear cells (BMMCs) obtained from patients with MM with 1q21 chromosomal aberrations (n = 3, E:T ratio 1:1). For each test group, the BMMCs were obtained from patients with distinct 1q21 aberrations. Post-coculture, the cytotoxic capabilities of FCRL5 CAR-T cells targeting CD138^+^ cells were assessed. Results include flow cytometry dot plots of a single exemplar from each cohort (left) along with an aggregated summary of the proportion of depleted CD138^+^ cells, represented as means ± standard deviations (right) (**P* < 0.05; ***P* < 0.01; ****P* < 0.001 vs. BCMA CARs group). **j** Assessment of TCM phenotype in CAR-T cells across repetitive cycles in multi-round co-culture experiments.


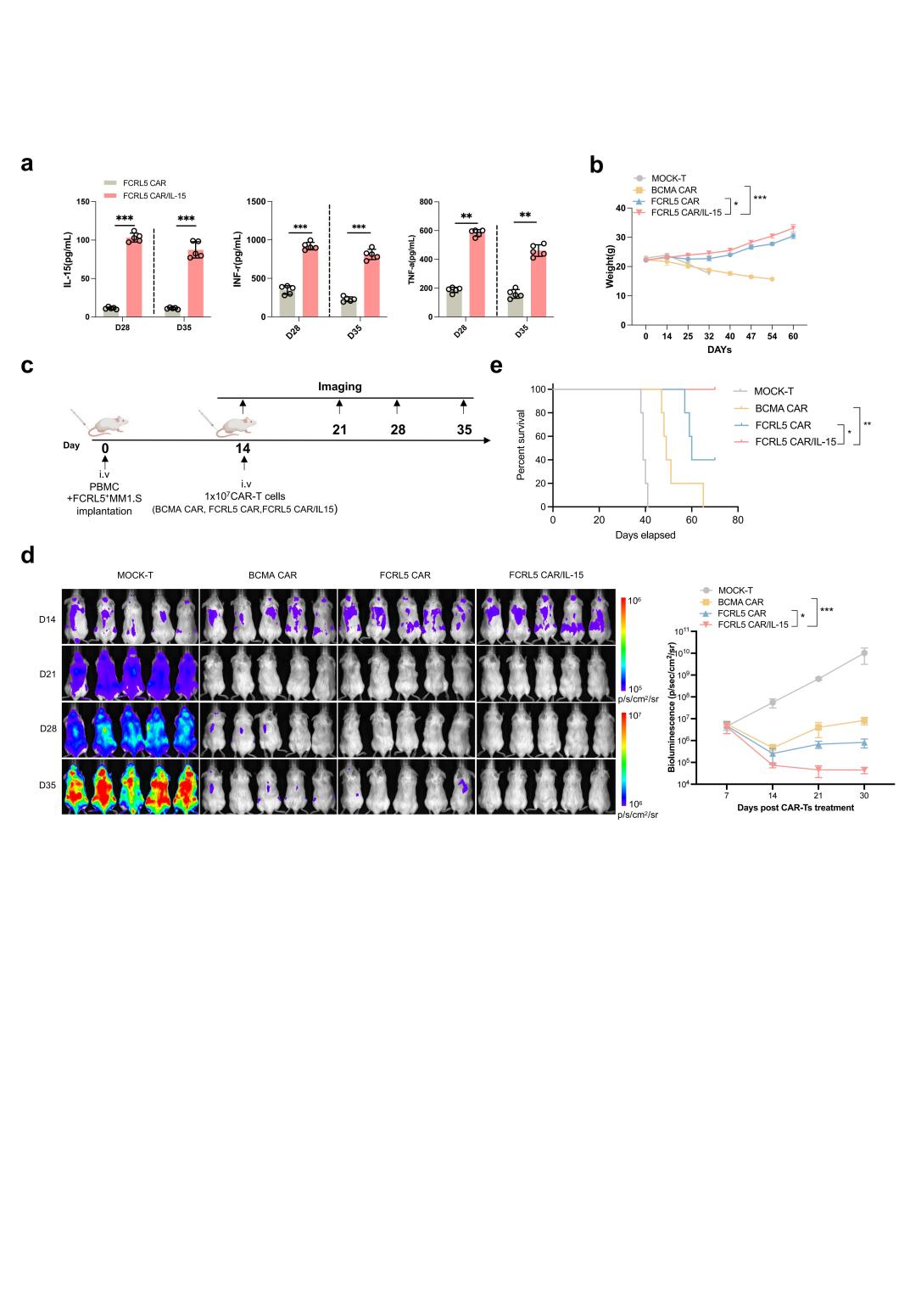


**Supplementary Fig. S5** Antitumor effects of FCRL5 CAR-T/IL-15 cells *in vivo*. **a** Elevated levels of IL-15 in the peripheral blood and higher secretion rates of the inflammatory cytokines IFN-γ and TNF-α quantitatively compared in FCRL5 CAR-T/IL-15-treated mice relative to FcRL5 CAR-T controls on days 28 and 35 (***P* < 0.01; ****P* < 0.001 vs. FCRL5 CARs group). **b** From day 14 onward, weight reduction was observed in the Mock T group compared with the treatment cohort. (***P* < 0.01 vs. Mock T group). **c** Schematic illustration showing how B-NDG model mice were treated with FCRL5-specific modified T cells. **d** Left: Representative bioluminescence images of xenograft models receiving assorted treatments across temporal intervals, with each group comprising five replicates (total B-NDG mice, n = 80). Right panel: Quantification of aggregate tumor flux, expressed in photons per second (p/s), conducted using Living Image software. **e** Log-rank test of Kaplan–Meier survival curves for tumor-bearing mice (**P* < 0.05; ***P* < 0.01; ****P* < 0.001 vs. BCMA CARs group).

| **Table S1a Clinical characteristics of patients in scRNA-seq discovery cohort** | | | | | | | | |
| --- | --- | --- | --- | --- | --- | --- | --- | --- |
|  | **patient id** | **Age** | **Gender** | **Race** | **ISS Stage** | **Treatment** | **TTPD in months** | **Treatment Status at time of collection** |
| WASHU Cohort 1 | 27522 | 69 | Male | White | 2 | KRD x 4; ASCT; IRD consolidation x 4 ; Ixa/Len maintenance | 32.73 |  |
|  | 27522_1 | 69 | Male | White | 2 |  |  | Primary |
|  | 27522_2 | 69 | Male | White | 2 | KRD x 3 |  | Remission-1 |
|  | 27522_3 | 69 | Male | White | 2 | KRD x 4 |  | Relapse-1 |
|  | 27522_4 | 69 | Male | White | 2 | ASCT |  | Relapse-2 |
|  | 27522_5 | 69 | Male | White | 2 | ASCT；IRD consolidation x 4 |  | Remission-2 |
|  | 27522_6 | 69 | Male | White | 2 | Ixa/Len maintenance |  | Relapse-3 |
|  | 77570 | 66 | Female | White | 1 | VRD-Daratumamab x 4; ASCT; VRD-Daratumamab x2; Dara/Len Maintenance | 34.34 |  |
|  | 83942 | 63 | Male | White | 3 | Unknown | NA |  |
| WASHU Cohort2 | MMY80649 | 62 | Male | White | 1 | KRD x 4; ASCT; Len main | 12.96 |  |
|  | MMY22933 | 62 | Male | White | 3 | Dara-MPR x 1; VRD x 4; Len maintenance | 10.89 |  |
|  | MMY22933 | 62 | Male | White | 3 | Dara-MPR x 1; VRD x 4; Len maintenance | 10.89 |  |
| Abreveations：Vel=Velcade；Rev=Revlimid；Dex=Dexamethasone；Dara=Daratumumab；Pom=Pomalidomide；Carf=Carfilzomib; KRD=Carfilzomib+Lenalidomide+Dexamethasone；IRD=Ixazomib+Lenalidomide+Dexamethasone；TTPD=Time To Progression of Disease； | | | | | | | | |
| \| **Table S1b Clinical characteristics of patients in 1Q21gain+/- discovery cohort** \| \| \| \| \| \| \| \| \| \| \| --- \| --- \| --- \| --- \| --- \| --- \| --- \| --- \| --- \| --- \| \| **Age** \| **Gender** \| **ISS Stage** \| **Chemo at time of biopsy** \| **Date of ASCT** \| **Collection Date** \| **Treatment Status at time of collection** \| **1Q21gain** \| **%CD38+FcRL5+** \| **Molecular analysis** \| \| 69 \| Female \| 1 \| NA \| NA \| 10.11.2022 \| NDMM \| Yes \| 22.88 \|  \| \| 62 \| Female \| 1 \| NA \| NA \| 10.19.2022 \| NDMM \| Yes \| 20.5 \|  \| \| 54 \| Male \| 2 \| NA \| NA \| 11.14.2022 \| NDMM \| Yes \| 25.4 \|  \| \| 67 \| Female \| 1 \| NA \| NA \| 2.22.2022 \| NDMM \| Yes \| 25.88 \|  \| \| 63 \| Male \| 3 \| NA \| NA \| 1.18.2022 \| NDMM \| Yes \| 30.5 \|  \| \| 71 \| Male \| 3 \| NA \| NA \| 11.24.2022 \| NDMM \| Yes \| 22.06 \| t(4;14),t(14;16) \| \| 68 \| Female \| 2 \| BRD \| NA \| 11.16.2022 \| Prev Treated \| Yes \| 36.06 \| RB1,D13S319 locus deletion \| \| 77 \| Male \| 1 \| BRD \| NA \| 10.14.2022 \| Prev Treated \| Yes \| 33 \|  \| \| 54 \| Male \| 3 \| CD38+BRD \| NA \| 10.19.2021 \| Prev Treated \| Yes \| 20.07 \| del7 ,t(4,14),TP53 \| \| 44 \| Female \| 3 \| BRD \| NA \| 12.23.2022 \| Prev Treated \| Yes \| 23.02 \|  \| \| 77 \| Male \| 3 \| NA \| NA \| 12.6.2022 \| NDMM \| NO \| 2.75 \|  \| \| 71 \| Male \| 3 \| NA \| NA \| 1.22.2022 \| NDMM \| NO \| 2.07 \|  \| \| 80 \| Female \| 3 \| NA \| NA \| 3.29.2023 \| NDMM \| NO \| 5.02 \| t(14;16) \| \| 82 \| Male \| 3 \| NA \| NA \| 3.22.2023 \| NDMM \| NO \| 3.13 \|  \| \| 54 \| Female \| 1 \| NA \| NA \| 3.24.2023 \| NDMM \| NO \| 1.05 \|  \| \| 59 \| Female \| 3 \| NA \| NA \| 12.6.2022 \| NDMM \| NO \| 7.75 \|  \| \| 79 \| Male \| 3 \| NA \| NA \| 14.12.2022 \| NDMM \| NO \| 69 \| t(14;16),P53 locus deletion \| \| 64 \| Female \| 1 \| NA \| NA \| 5.15.2023 \| NDMM \| NO \| 11.4 \|  \| \| 74 \| Female \| 2 \| NA \| NA \| 10.31.2021 \| NDMM \| NO \| 12.75 \|  \| \| 47 \| Male \| 2 \| NA \| NA \| 2.16.2022 \| NDMM \| NO \| 10.05 \|  \| \| Abreveations：BRD=Bortezomib+Lenalidomide+Dexamethasone；Prev =Previously；NDMM=Newly Diagnosed Multiple Myeloma；ASCT=Autologous Stem；  CD38=anti-CD38 monocloning antibody Cell Transplantation \| \| \| \| \| \| \| \| \| \| | | | | | | | | |
